# SBRC-Nottingham: sustainable routes to platform chemicals from C1 waste gases

**DOI:** 10.1042/BST20160010

**Published:** 2016-06-09

**Authors:** Alan Burbidge, Nigel P. Minton

**Affiliations:** * BBSCR/EPSRC Synthetic Biology Research Centre (SBRC), The University of Nottingham, University Park, Nottingham, NG7 2RD, U.K.

**Keywords:** C1 gas, gas fermentation, industrial biotechnology, platform chemicals, synthetic biology

## Abstract

Synthetic Biology Research Centre (SBRC)-Nottingham (www.sbrc-nottingham.ac.uk) was one of the first three U.K. university-based SBRCs to be funded by the Biotechnology and Biological Sciences Research Council (BBSRC) and Engineering and Physical Sciences Research Council (EPSRC) as part of the recommendations made in the U.K.'s Synthetic Biology Roadmap. It was established in 2014 and builds on the pioneering work of the Clostridia Research Group (CRG) who have previously developed a range of gene tools for the modification of clostridial genomes. The SBRC is primarily focussed on the conversion of single carbon waste gases into platform chemicals with a particular emphasis on the use of the aerobic chassis *Cupriavidus necator*.

## Background to the CRG

Established in 2004, the Clostridia Research Group (CRG) have previously developed a range of gene tools for the modification of clostridial genomes [1–9]. These have been used to conduct research on biofuels [10–13], *Clostridium difficile* infection and control [14–17], understanding the food pathogen *Clostridium botulinum* [18–21] as well as developing therapeutic strategies for treating solid tumours [22–25]. The majority of this work has focussed on clostridia but the establishment of the Synthetic Biology Research Centre (SBRC)-Nottingham has opened up the opportunity to work on a broader spectrum of bacteria and to now include aerobes, specifically *Cupriavidus necator*.

## CRG roadmap technologies

The CRG have developed a series of technologies which when sequentially applied; have allowed the modification of new clostridial hosts. Several of the components of this ‘roadmap to gene system development’ are subject to patent protection: Synthetic Operon Construction in Clostridia Allele Coupled Exchange (ACE, WO 2009/101400); A negative/counter selection marker for use in clostridia: CodA selectable marker (WO2010/084349); Transposon delivery system for clostridia (vector) TraDis Transposon system (WO2013/144653); Orthogonal bacterial expression system (WO2013/144647); ClosTron (www.clostron.com) technology (WO/2007/148091). These technologies, along with the pMTL80000 modular vector series [1], are used to facilitate gene transfer and gene knockout/knockin and to generate bespoke chassis carrying genome inserted synthetic pathways and application specific modules.

## SBRC focus

Unlike many of the other U.K. SBRC's, the scope of the SBRC-Nottingham is highly directed and focuses on the conversion of waste one-carbon (C1) gases through bacterial fermentation to industrially useful hydrocarbon-based chemicals. The particular focuses are those with multiple bonds (alkenes) such as 3-hydroxyproprionic acid, ethylene, propylene, isobutene, butadiene and isoprene. The molecules were chosen because of their industrial relevance, because they are ‘stepping off’ points for the production of more complex compounds using sustainable chemistry and because they are volatile, hence more easily recovered from the fermentation system which in turn helps prevent products accumulating to levels that are toxic to the bacteria.

The long-term aim of the SBRC-Nottingham is to break our reliance on fossil fuels as the source of the chemicals we use in our everyday lives and to make these chemicals sustainably using waste C1 while at the same time reducing C1 gas evolution to the atmosphere, hence reducing greenhouse gas emissions.

The SBRC-Nottingham is applying learning from the CRG to the Gram-negative bacterium *C. necator* (formerly known as *Ralstonia eutropha* or *Alcaligenes eutropha*). This industrially relevant, facultatively chemolithoautotrophic bacterium is able to grow with organic substrates or H_2_ and CO_2_ under aerobic conditions. Under nutrient limitation, *C. necator* directs the majority of its reduced carbon flux into the synthesis of poly[(*R*)-3-hydroxybutyrate] (PHB), a biopolymer stored in intracellular granules. When cultivated with H_2_ and CO_2_, *C. necator* forms up to 61 g/l of PHB in 48 h, representing ∼70% of total cell weight [26]. Mutants defective in PHB production secrete large amounts of pyruvate into the growth medium when cultured chemolithoautotrophically, suggesting that these mutants maintain a comparable carbon flux in the presence or absence of PHB biosynthesis [27,28].

Biosynthesis of PHB under nutrient-limiting conditions in *C. necator* has been industrially exploited in the production of biodegradable plastic for more than a decade [e.g. by Imperial Chemical Industries (ICI)/Zeneca, Monsanto and Metabolix]. Only recently has *C. necator* been recognized as a candidate for biofuel production from CO_2_, potentially producing carbon-neutral biofuels from non-photosynthetic sources. Li and colleagues engineered a *C. necator* strain for electromicrobial conversion of CO_2_ to C_4_ and C_5_ alcohols [29]. In their system, electricity powered the electrochemical reduction of CO_2_ at a cathode to produce formate, which was then converted into isobutanol and 3-methyl-1-butanol by an engineered *C. necator* strain. With such a capacity to synthesize and store fixed carbon from CO_2_, *C. necator* offers the promise of a chassis which through synthetic biology approaches will be able to synthesize and store other, industrially important platform chemicals.

## Interdisciplinarity

Synthetic biology is inherently multidisciplinary and the Centre's academic leadership team membership is drawn from faculties and schools across the university including Chemistry, Life Sciences, Mathematics, Computer Science, Engineering, Social Sciences, Pharmacy and Biosciences. To create a coherent research community, the SBRC-Nottingham has been provided with a dedicated floor in the University's flagship Biomolecular Sciences research building on the University Park campus. This is a £40 million multidisciplinary research facility which also houses other multidisciplinary teams. Researchers within the SBRC-Nottingham are co-located, in particular ‘lab-based’ scientists and mathematical modellers work in multidisciplinary teams. The SBRC-Nottingham is located within the science and engineering hub of the university with mechanisms in place for researchers based on other campuses to spend dedicated time within the team. The SBRC-Nottingham positions Responsible Research and Innovation central to its activities with social scientists physically based within the SBRC to maximize interaction with synthetic scientists. There is a strong outreach ethos with many links between the SBRC-Nottingham, local schools, Café Scientifique, Pub PhD, the media and other U.K. SBRCs. Many of these activities are undertaken in partnership with C1net (www.c1net.co.uk), a Biotechnology and Biological Sciences Research Council (BBSRC) Network in Industrial Biotechnology and Bioenergy (NIBB) coordinated from Nottingham.

## Equipment and facilities

The SBRC-Nottingham has a wealth of dedicated new equipment, including flow cytometry, lab-scale fermentation facilities, analytics including HPLC, GC, MS, Raman spectroscopy as well as near- and mid-IR spectroscopy. A robotics suite is being established with bespoke systems able to carry out automated gene assembly, transformations, culturing and clone selection. The fermentation systems are of particular note. A gas fermentation laboratory was first established in 2013 to explore anaerobic gas fermentation. Building on the knowledge gained from this facility, a new laboratory suite dedicated to anaerobic and aerobic gas fermentation for the SBRC has been commissioned and when fully operational will comprise over 20 gas-enabled Controlled Stirred Tank Reactors (CSTRs) as well as a gas-enabled RoboLector®, benchtop micro fermentation system that allows high-throughput 48×2 ml fermentations together with online monitoring. These facilities are among the few available in the U.K. which have the capability to ferment waste C1 gases into fixed carbon from microbes. Although the facility operates only at lab-scale, the new knowledge being generated will critically inform demonstrator and scale-up fermentation research. We are linking up with the Centre for Process Innovation on Teesside, U.K. to establish best practice in gas fermentation and gas safety in the U.K. and to share experience so that lab-scale fermentation knowledge more readily integrates with scale-up to larger plant.

## Training

The University has a BBSRC Doctoral Training Programme which annually recruits 50 students or which a third are targeted for Industrial Biotechnology, an University funded SBRC Synthetic Biology Doctoral Training Programme with 25 studentships to be recruited over the 5-year funding lifetime of the SBRC and an Engineering and Physical Sciences Research Council (EPSRC) Doctoral Training Centre in Sustainable Chemistry which will recruit 60 students over the same time period. Together, these programmes are training the next generation of multidisciplinary scientists in Synthetic Biology and related areas of science.

## Industrialization and application

SBRC-Nottingham is carrying out fundamental academic research to understand the physiology and metabolism of microbial chassis that could have industrial application. Key outputs of the centre are (i) knowledge of fermentation conditions and constraints, (ii) metabolic models which have through iteration led to and (iii) optimized production chassis. Many of these outputs will be of interest to industry either directly or to inform industry's processes which are developing in parallel. Translation to industry and the wider academic community is further facilitated through the activities of the Nottingham–led NIBB, C1net (www.c1net.co.uk).

Without compromising the SBRC-Nottingham's academic interests and without constraining the widespread distribution of project outputs, we have teamed up with a newly formed synthetic biology company called CHAIN Biotechnology. This company provides one of the routes we are using to facilitate the wider adoption and also the exploitation of the outputs from the SBRC-Nottingham. This company has already taken responsibility for the distribution of SBRC background vectors (http://chainbiotech.com/modular-plasmids/) and intellectual property (IP). Through the University of Nottingham, over 250 MTAs have been put in place for the pMTL80000 modular vector series developed by the CRG [1]. CHAIN Biotechnology will further expand the distribution of these materials and build a global community of users to maximize the wider impact of SBRC-Nottingham research outputs. The pMTL80000 series is designed for use in clostridial chassis. Further series are under development for *Cupriavidus* (pMTL70000 series), *Geobacillus* (pMTL60000 series) and for methanotrophs (pMTL90000 series).

## Abbreviations

BBSRC: Biotechnology and Biological Sciences Research Council
C1: one-carbon
CRG: Clostridia Research Group
EPSRC: Engineering and Physical Sciences Research Council
ICI: Imperial Chemical Industries
IP: intellectual property
MTA: Material Transfer Agreement
NIBB: Network in Industrial Biotechnology and Bioenergy
PHB: poly[(*R*)-3-hydroxybutyrate]
SBRC: Synthetic Biology Research Centre
